# Factors Associated with Depression among the Elderly Living in Urban Vietnam

**DOI:** 10.1155/2018/2370284

**Published:** 2018-11-25

**Authors:** An T. M. Dao, Van T. Nguyen, Huy V. Nguyen, Lien T. K. Nguyen

**Affiliations:** ^1^Department of Epidemiology, Institute for Preventive Medicine and Public Health (IPMPH), Hanoi Medical University, 01 Ton That Tung Str., Dong Da District, Hanoi, Vietnam; ^2^Quality Management Department, Dong Da General Hospital, 192 Nguyen Luong Bang Str., Dong Da District, Hanoi, Vietnam; ^3^Department of Health Management and Organization, Institute for Preventive Medicine and Public Health, Hanoi Medical University, 01 Ton That Tung Str., Dong Da District, Hanoi, Vietnam; ^4^Rehabilitation Center, Bach Mai Hospital, 78 Giai Phong Str., Dong Da District, Hanoi, Vietnam; ^5^Rehabilitation Department, Hanoi Medical University, 01 Ton That Tung Str., Dong Da District, Hanoi, Vietnam

## Abstract

The proportion of elderly people in big cities of developing countries, including Vietnam, is rapidly increasing during the age of rampant urbanization. This is being followed by a sustained rise of illnesses, especially mental health issues. The objective of this study was to analyze the association between depression and the factors associated with depression among the elderly. In a cross-sectional study, 299 elderly living in Hanoi, Vietnam, were approached for data collection. Self-reported depression among the elderly was 66.9% (32.8% mild, 30.4% moderate, and 3.7% severe cases). In multivariate analysis, there were significant associations between age, number of physical activities, number of medicine intake, and 3 domains of quality of life (physical health, psychological health, and environmental health) and depression. Age and the number of medicine intake are positively correlated with depression, accounting for 57.94% and 58.93%, respectively. On the contrary, the number of physical activities and the 3 domains of quality life mentioned above are negatively correlated with depression. In the urban setting of a developing country like Vietnam, the elderly have experienced common depression. Recognizing depression among the elderly—which is individual and social—helps us design public health programs. Screening for early depression, joining social programming, and participating in physical activities may improve the mental life of the elderly.

## 1. Introduction

Depression is a common mental disorder that presents with depressed mood, loss of interest or pleasure, feelings of guilt or low self-worth, disturbed sleep or appetite, low energy, and poor concentration [1]. The global point, one-year, and lifetime prevalence of depression are 12.9%, 7.2%, and 10.8%, respectively [2]. The average total cost of patients with depression is US$7,638 per patient-year and indirect costs (e.g., unemployment and loss of productivity) dominated the total costs [3]. Risk factors for geriatric depression include poor health, brain injury, low folate, and vitamin B12 and raised plasma homocysteine levels [4]. Elderly who died of suicide and had a past history of suicidal behavior were more likely to suffer from depression [5]. These problems can become chronic or recurrent and lead to substantial impairments in an individual's ability to take care of his or her everyday responsibilities. Depression is the leading cause of disability as measured by Years Lived with Disability (YLDs) and the 4th leading contributor to the global burden of disease measured by the Disability Adjusted Life Years (DALYs) in 2000. By the year 2020, if current trends for demographic and epidemiological transition continue, the burden of depression will increase to 5.7% of the total burden of disease [1].

Depression results from the complex interaction of biologic predisposition and life events or the person's social and internal world as a potent source of depressive risk over the lifespan [6]. At the biologic predisposition, genetics had been reported a major attribution to depression, especially to major depression [7, 8]. In terms of person's social and internal psyche, there are many factors which indicated having association with depression: physical activities, medical illness, quality of life, social connectedness, drug, and alcohol [9–19]. Although depression is not a normal part of ageing, it is a true and treatable medical condition, but older adults still are at an increased risk for experiencing depression. However, healthcare providers may mistake an older adult's symptoms of depression as just a natural reaction to illness or the life changes that may occur as we age and therefore not see the depression as something to be treated. Older adults themselves often share this belief and do not seek help because they do not understand that they could feel better with appropriate treatment [20]. In the elderly, the association between depression and chronic illnesses is explained by accompanying poor self-reported health and functional status [21].

In Vietnam, people aged from 60 years or above are defined as older persons or elderly people (Clause 1, Chapter 1, The Ordinance on Elderly). The statistics from the 2009 Vietnam population and housing census as well as Vietnam Household Living Standard Survey in recent years show that the proportion of old people is rising sharply, like in almost every country in the world. Vietnam is not an exception. The number of elderly people increases yearly since 2008 when the elderly population represented 11% of the Vietnamese population. The age groups 60-64 and 65-69 are the most concerned by the ageing of the population [22].

Depression is one of four major noncommunicable diseases (2.8% of the population), it increases with age and is higher in the big cities [22]. According to Professor Hoang Moc Lan, Psychology Department, University of Social Sciences and Humanities at Vietnam National University, depression increases among those living independently. Forty-seven percent of the sample in Da Nang city—a popular city for tourism in Vietnam—had scores above the cut-off for clinical depression [23]. However, there was no available data on depression among elderly who are living in Hanoi City, the second biggest city in Vietnam to date.

To estimate the extent of depression among elderly living in Hanoi and determinants of depression in a Vietnamese context in order to inform evidence based policy for preventing depression among elderly, this pilot study aims at measuring prevalence of depression and analyzing any associations between physical activities, morbidities, quality of life, social connectedness, and depression among the elderly. Additionally, recommendations for improving scales measuring associated factors will be formulated.

## 2. Methods

### 2.1. Study Design and Setting

This cross-sectional study was conducted in Trung Tu commune, Ha Noi city, which is located in Northern Vietnam. This commune has one of the densest populations in Ha Noi and is mainly comprised of government officers who live in 62 dormitories and 2 residential districts with convenient transportation and close proximity to entertainment venues, national hospitals, and schools. Until 2011, there were 1,593 elderly people in Trung Tu, accounting for 11.78% of the total population of the commune.

### 2.2. Study Subjects

This is a cross-sectional study based on a mental health screening pilot program among the elderly in one urban commune in Hanoi City, as a component of a larger annual health assessment event provided by the commune. Elderly who were 60 years old and above and who had been living in Trung Tu commune for at least 1 year voluntarily registered to participate in the mental health screening day and were recruited to participate in filling out a self-administered questionnaire which measure depression and associated factors.

### 2.3. Sample Size and Data Collection

In mid-2012, three hundred (6%) out of 5,000 elderly living in Trung Tu commune were recruited for a pilot mental health screening day through their voluntary registration. Announcements regarding the mental health screening were written on boards at dwelling areas with a toll-free number. Health workers screened callers to study eligibility and made a consecutive recruitment of the first 300 callers. Based on the list of elderly identified through their voluntary registrations and conveniently recruited, collaborators contacted the registering people at their houses and gave them consent forms. Participants received a self-administered questionnaire from research assistants immediately following informed consent. The self-administered questionnaire included one section measuring the signs and symptoms of depression and 4 other sections focusing on physical activities, morbidities, quality of life, and social connectedness.

### 2.4. Measures

#### 2.4.1. Dependent Variable “Depression”

The 1965-Zung self-rating depression scale (Zung SDS) was used. The original form of the Zung SDS contains 20 questions asking about feeling levels of elderly on (1) depressed mood, (2) morning symptoms, (3) crying, (4) insomnia, (5) diminished appetite, (6) weight loss, (7) sexual interest, (8) constipation, (9) palpitations, (10) fatigue, (11) clouded reasoning, (12) difficulty with completing tasks, (13) difficult decision making, (14) restlessness, (15) lack of hope, (16) irritability, (17) diminished self-esteem, (18) life satisfaction, (19) suicidal ideation, and (20) anhedonia. For each question, the study subject rates are 1 = a little of the time, 2 = some of the time, 3 = a good part of the time, or 4 = most of the time.

Raw score of depression was calculated by total score of 20 questions asked using Dung SDS and then was converted into a 100-point scale (SDS Index) using equation: SDS Index = (Raw Score/80 total points) x 100. This SDS Index was then interpreted into levels of depression as follows:

- Score <50: normal
- Score 50 - 59: mild depression
- Score 60 - 69: moderate or marked major depression
- Score ≥70: severe or extreme major depression

#### 2.4.2. Independent Variables

*Demographic characteristics* included age, sex, marital status, education, job, and living status.

*Physical activities* were measured by the PASE scale [24]. The PASE uses a 4-point Likert scale in which 0 is never, 1 is “a little of the time”, 2 is “some of the time”, 3 is “good part of the time”, and 4 is “most of the time”. The physical activity variable was defined as a sum score of these 5 questions which would range from 0 to 20.

*Smoking status*: “have you smoked in the past 12 months?” (“yes” coded “1” and “no” coded “0”).

*Drinking behaviors*: “have you drunk alcoholic beverages (spirits or beer) in the past 12 months?” (“yes” coded “1” and “no” coded “0”).

*Medication use* (proxy of morbidity and behaviors): referencing from Scales of the WHO STEPwise approach to chronic disease risk factor surveillance-Instrument V2.1 [25]. It includes 21 questions with answer “yes” coded “1” and “no” coded “0”. Medicine intake variable was then defined as the total number of drug groups that one elderly has taken in the last 12 months. This variable has a value scoring from 0 to 21.

*Social connectedness* was measured by the Short version of Adapted Social Capital Assessment Tool (SASCAT) including 3 components with 18 items asked using scale “yes” or “no” [25].* Social connectedness* variable was defined as a sum score of these 18 questions which would ranges from 0 to 18 score.

*Quality of life* was measured by WHOQoL-Brief [26] including 26 items of 4 components which were asked with Likert scale from 1-5.

### 2.5. Statistical Analysis

Data analysis was performed with statistical software Stata version 12. Descriptive and multivariate methods were used to examine depression. Descriptive statistics were performed initially to describe the distribution of sample demographic characteristics, physical activities, smoking status, drinking behavior, medication use, social connectedness, and quality of life. The difference in depression between two or more groups was analyzed by Skewness-Kurtosis test, Mann-Whitney test, T-test, and Kruskal Wallis test. The factors associated with depression were detected with multivariate models, using stepwise multiple regression analysis.

### 2.6. Ethical Statement

The study was accepted and approved by the Ethical Committee of the School of Public Health in May 2012 at ethical clearance no. 012-111/DD-YTCC. The participants were informed of key contents and objectives of the research so they were voluntary to participate and that they could refuse to answer any question which made them uncomfortable or could withdrawal at any time without it having any effect on them in any way. The individual information of participants was kept confidential and not used in the data analysis. The results are just for research purposes and not used for any other purpose.

## 3. Results

### 3.1. Selected Characteristics of the Sample

Out of 300 elderly people who were recruited, one individual did not meet inclusion criteria, and the characteristics of the remaining 299 participants are shown in Table 1. The proportion of males to females was balanced at 48.8% and 51.2%, respectively. The mean age of study participants was 70.6 years, with no statistical difference between the mean age of males and females (p>0.05). The proportion of subjects aged 70 years plus was 54.5% with no difference between males and females regarding their age groups. The majority of participants (72.2%) completed a high school education, with significantly more males having completed higher education than females (p>0.05). Most of them were working as government officers (80.3%), married (84.6%), and living primarily with their husband or wife or children or relatives (96%).

### 3.2. Depression among the Elderly

Table 2 showed the distribution of depression according to demographic, life styles (physical activities, smoking, and alcohol), medicine intake, quality of life, and social connections. 66.9% of elderly people had risks of slight, moderate, and severe depression at 32.8%, 30.4%, and 3.7%, respectively. The distribution of depression was different according to age group, alcohol use, physical activity, medicine intake, quality of life, and some components of social connectedness (participating in social activities and social connectedness). Those aged 70 and older had a statistically significantly higher proportion of depression than the group under 70 years old regarding every level of depression, from mild (36.2% versus 28.7%) and moderate (34.4% versus 25.7%) to severe (4.9% versus 2.2%). Depression was distributed higher and more severely in the group that was not using alcohol than in the group that has used alcohol regarding levels of depression from normal, mild, moderate, and severe (28.6%, 25.2%, 39.5%, and 6.7%, respectively). The physical activity participation showed that depression significantly decreased when physical activity increased (p <0.05). There was no association to depression if the elderly participated on average in more than 3 out of 5 physical activities on the survey; there was slight depression if the elderly participated in average in 3 activities, moderate depression when the elderly participated in average of 2 physical activities, and heavy depression when practiced in average in 1 activity. There were significant differences (p <0. 05) in frequency of medicine intake among the elderly. Results showed that, among the elderly who did not use medication or vitamins, 50% of them suffered from depression (30% was light, 20% moderate and none severe). Among the elderly who used 1 to 5 types of medicine, 50.8% had depression (26.2% mild, 23.1% medium, and 1.5% severe). The use of 6-10 drugs yielded 64.8% depression (33.3% mild, 29.5% moderate, and 1.9% severe). Those who used more than 10 drugs had a 79% prevalence of depression (36.1% mild, 36.1% moderate, and 6.8% severe). A significant decrease in depression was observed as QoL scores increased (p<0.001). There was no depression in any of the 4 QoL domains if the score was over 60 points, while on average QoL scores below 47 points, in any of the 4 domains, were often associated with severe depression. There were significant differences (p <0.05) in depression depending on frequency of social connectedness, measured by social activity participation and connectedness to others. Results showed that when social connectedness score decreased, the reports of depression increased. Out of the elderly who participated in less than 3 connectedness activities, 68.3% had depression (25.1% mild, 37.2% moderate, and 6.0% severe) while those who participated in 3 and more connectedness activities get 64.6% depression (44.8% mild, 19.8% moderate, and no severe cases identified). A connectedness score under 4 was associated with a depression prevalence of 73.8% (30% mild, 35% moderate, and 8.8% severe cases), while those who have a connectedness to others score of 4 and above get 64.4% depression (33.8% mild, 28.8% moderate, and only 1.8% severe cases). There were no differences in distribution of depression by gender, levels of education, occupation, marital status, living arrangement, tobacco use, smoking status, and receiving support.

### 3.3. Factors Associated with Depression among the Elderly

Table 3 presented the univariate and multivariate analysis factors associated with depression. In the univariate analysis, age, participation in physical activities, medicine intake for health problems, alcohol use, quality of life, and social connectedness were associated factors with depression. When age, physical participation, or medicine intake increased by 1 unit, there was a statistically significant decrease in depression by 0.37, 3.27, and 0.46 units, respectively. Depression decreased 3.4 times among elderly who drank alcohol compare with those who did not use. When physical quality of life, spiritual quality of life, social quality of life, environmental quality of life, and social connectedness increased by 1 unit, depression decreased significantly by 0.44, 0.5, 0.28, 0.44, and 0.76 units, respectively.

In the multivariate analysis, both models (described in Methods section) showed that there were significant associations between age, number of physical activities, number of medicine intake, 3 domains of quality of life (PH_QoL, PsH_QoL), and depression with the extent of influence at 57.94% and 58.93% for age and number of medicine intake, which are positively correlated to depression, while the number of physical activities and 3 domains of quality of life were correlated with depression in a reverse way. Moreover, model 2 showed that there were 3 more specific social factors including household composition, participating in a religious group, and participating in discussions with neighbors about their communities' issues which have also significant association with depression.

## 4. Discussion

The current data indicates that 66.89% of the elderly living in the Trung Tu commune had risk of depression from mild and moderate to severe levels at 33.11%, 32.78%, and 3.68%, respectively. Our assessment of severe depression at 3.38% aligns with the prevalence of severe depression (3-6%) clinically diagnosed at mental health hospitals [27, 28]. The results indicated that the elderly seem to be diagnosed only at a late stage of depression, while those who get mild and moderate depression have not been screened and diagnosed yet, and they would be diagnosed with depression and hospitalized only when their depression would have progressed to a severe form [29]. This study indicates a high proportion of depression among the elderly, which is also pointed out by several other studies, with results ranging from 30.1% in a study of 418 elderly in Malaysia [30] to 40% in a study of 3,840 individuals aged 50 years or above in South Africa [14]. Therefore, findings of this current study suggest that, in the perspective of prevention, one should routinely screen for depression using questionnaire tools for the elderly living in the community, to detect depression early rather than letting them be diagnosed at very late stages, in order to reduce disease burden for their families and society.

Regarding factors associated with depression, both model 1 and model 2 in Table 3 showed that there was a significant association between age, number of physical activities, number of medicine intake, 3 domains of quality of life (PH_QoL, PsH_QoL), and depression with the predictive meaning of influence at 57.94% and 58.93% for age and number of medicine intake, which are positively correlated to depression, while the number of physical activities and 3 domains of quality of life were correlated with depression in a reverse way. From the physical health viewpoint, depression in the elderly was found to be associated with increased levels of proinflammatory cytokines including interleukin- (IL-) 1B and IL-6 [31]. These cytokines are associated with chronic medical conditions including rheumatoid arthritis and cardiovascular diseases [32]. In order to improve physical, psychological and environmental health, horticultural therapy, which involves gardening environment and physical activity, was found to reduce IL-6 levels and enhance psychological well-being of the elderly [33]. Many other studies also indicated association of these factors with depression. In a study of 3,840 individuals aged 50 years old or above in South Africa, multivariable logistic regression showed that functional disability, quality of life, and chronic conditions were strongly associated with past 12 months of depression [14]. In a study of 2,808 older adults in Singapore, logistic regression at baseline showed that age, number of medical problems, and health activities were associated with depression. In addition to that, depression was also associated with baseline ADL, baseline MMSE, living alone versus with others, lonely versus not lonely and living arrangement loneliness, at follow up, health activities score, baseline GDS, and lonely status [34]. In a study of 696 elderly in North Carolina, multivariable regression showed that sex, formal education, living arrangement, number of prescription medications, and Physical Component Summary were associated with depression; however we did not match these results [35]. Another difference with our study can be found in a study of 150 elderly persons from Sharqia city, which showed that correlation between Depression and Environment QoL is not significant and correlation between Depression and Physical QoL, Psychological QoL, and Social QoL is significant [36]. Moreover, model 2 in Table 3 showed that there were 3 more specific social factors including living arrangement, participating in a religion group, and participating in discussions with neighbors about their communities' issues which also have a significant association with depression. Other differences come from studies by A Rashid and Wooksoo Kim, where multivariable regression showed that occupation, income, age, education, health status, health concerns, health promotion variables, and religion were significantly associated factors with depression [30, 37].

Culture of Asian family models could give some insight to why these 3 factors have associations with depression among the elderly. In Asian countries like Vietnam, due to multiple generations living together, grandparents and parents often live with descendants, with the imbedded meaning that “youth is taken cared by his/her mother and father and elderly are taken cared by their offspring”. On the other hand, family connectedness plays an important role in mental and physical health of the elderly in Asian countries. This might be a little different between Asia countries and the Western lifestyle where young people over 18 would leave their home and parents for their independence, and the elderly may live the rest of their lives in a pension home with support from social welfare of the state, easier and better access to services. They would bear the impact of social cohesion rather than families. Therefore, the elderly may live independently from their relatives without much influence to their mental health. In the United States, between 2002 and 2012, private-pay prices for a private or semiprivate room in a nursing home grew by an average of 4.0 percent and 4.5 percent per year, respectively, and in 2012, there were 1.4 million people in nursing homes in the US [38]. Therefore, many studies focusing on European and Western countries showed the link between depression and social connectedness, participation, and integration in creating and maintaining elder-friendly communities. These studies also indicate that the relationship between social disconnectedness and mental health works at least in part through perceived isolation [39, 40]. Additionally, religion can play a significant role in mental health of the elderly. Many Vietnamese follow Buddhism, which means that, after retirement, the elderly live away from “business life”, spending their time to find tranquility and health. In a review of 444 studies, 272 (61%) studies reported significant inverse relationships with depression and 28 (6%) found relationships between religion/spirituality (R/S) and greater depression [41]. An independent review by Timothy B. Smith in 2003 found that out of 147 studies involving 98,975 subjects, the average correlation between R/S and depression was −0.10 [40]. Moreover, in Asian culture, village culture seems to dominate in all communities. Especially in Vietnam, where people live not only for individual well-being but also for community well-being in the meaning that people should be a part of and have a certain role in their communities, village culture has created favorable conditions for the elderly to be “immersed” in the community. Other studies showed that participation in social activity, such as a neighborhood association, retired or elderly association, or charitable association, alleviates depressive symptoms [39, 42]. Hyun Woong Roh also made recommendations: participation in physical, social, and religious activity was associated with a decreased risk of depression in the elderly, and that risk of depression was much lower in the elderly who participated in two or three of the above-mentioned types of activity than those who did not [16].

However, our current study indicates that social connectedness has no association with depression. Perhaps connectedness is not associated with mental health of the elderly within Vietnamese setting because after retirement, many live a life limited to their own families without participating in social works. Social work is still a young field in Vietnam and has not been developed in an academic setting until recently. Therefore, social programming may not be as prevalent as in other countries. In a study by The Irish Association of Social Workers, the Special Interest Group on Ageing showed that the role of Social Workers increased the quality of material life of the elderly; improved the social support and protection policies towards the minimum living standard for the elderly; developed and improved the quality of service system and care facilities for the elderly; and attached special importance to elderly people with disabilities, elderly people who are poor people without supportive people, and elderly people from ethnic minorities. On the other hand, at the age of 75 or older, most elderly people are incapable of working, often with illness, and are significantly older than life expectancy. Therefore, it is necessary to lower the age to receive social support for the elderly, which is currently 75 years [43]. Therefore, social work training should be emphasized in the coming years. After retirement, people should participate in social activities so that they can reduce the influence of negative social factors like isolation on mental health, especially depression.

## 5. Conclusion

The study highlighted several key areas for improving mental health among the elderly in inner cities of developing countries such as Vietnam. It is crucial to provide screening for early depression by using questionnaire tools routinely for the elderly living in the community, to detect depression early rather than letting them be diagnosed at very late stages, in order to reduce disease burden for their families and society. The elderly should be encouraged and supported in engaging in physical activities in order to improve health. Enhancing physical health, psychological health, and environmental health of elderlies would also be helpful. Old people are expected to live with their family or relatives, participating in religious activities and group communities. Social work training could be emphasized in the coming years to create programs for people to participate in social activities after retirement.

## Figures and Tables

**Table 1:** Selected sociodemographic characteristics.

**Contents**	**Total**	**Male**	**Female**	**p-value**
	**n (**%**)**	**n (**%**)**	**n (**%**)**	
**Total**	**299 (100)**	**146 (48.8)**	**153 (51.2)**	
**Mean ± Sd (age)**	70,6 ± 7.4	71,7 ± 7.9	69,59 ± 6,84	0.1
**Age group**				
60-69	136 (45.5)	62 (42.5)	74 (48.4)	0.3
70 - 91	163 (54.5)	84 (57.5)	79 (51.6)	
**Education**				
Under & high school	83 (27.8)	32 (21.9)	51 (33.3)	**0.03**
Over high school	216 (72.2)	114 (78.1)	102 (66.7)	
Occupation				
Government officers	240 (80.3)	119 (81.5)	121 (79.1)	0.6
Others	59(19.7)	27 (18.5)	32 (20.9)	
**Marital status**				
Single	46 (15.4)	9 (6.2)	37 (24.2)	0.001
Married	253 (84.6)	137 (93.8)	116 (75.8)	
**Living arrangement**				
Alone	12 (4.0)	3 (2.1)	9 (5.9)	0.1
Family or others	287 (96.0)	143 (98.0)	144 (94.1)	

**Table 2:** Distribution of depression by demographical and social characteristics.

**Contents**		**n**	**No depression**	**Depression**			**p-value**
			**Normal**	**Slight depression**	**Moderate depression**	**Severe depression**	
			**n(**%**)**	**n(**%**)**	**n(**%**)**	**n(**%**)**	
**Proportion depression**		**299**	**99 (33.1)**	**98 (32.8)**	**91 (30.4)**	**11 (3.7)**	
**Demographic characteristics**							
Age group	60 - 69	136	59 (43.4)	39 (28.7)	35 (25.7)	3 (2.2)	**0.01**
	70 - 91	163	40 (24.5)	59 (36.2)	56 (34.4)	8 (4.9)	
Sex	Male	146	47 (32.2)	50 (34.2)	43 (29.5)	6 (4.1)	0.92
	Female	153	52 (34.0)	48 (31.4)	48 (31.4)	5 (3.2)	
Education	Over high school	83	27 (32.5)	22 (26.5)	32 (38.6)	2 (2.4)	0.22
	Lower or high school	216	72 (33.3)	76 (35.2)	59 (27.3)	9 (4.2)	
Occupation	Government officers	240	85 (35.4)	77 (32.1)	71 (29.6)	7 (2.9)	0.23
	Others	59	14 (23.7)	21 (35.6)	20 (33.9)	4 (6.8)	
Marital status	Single	46	14 (30.4)	21 (45.7)	8 (17.4)	3 (6.5)	0.07
	Married	253	85 (33.6)	77 (30.4)	83 (32.8)	8 (3.2)	
Living arrangement	Alone	12	3 (25.0)	3 (25.0)	5 (41.7)	1 (8.3)	0.63
	Family or others	287	96 (33.4)	95 (33.1)	86 (30.0)	10 (3.5)	
**The amount of physical activities**			3.48 ± 1.14	2.63 ± 1.29	2.15 ± 1.14	1.0 ± 1	**0.001**
**Behaviors**							
Smoking status	Absent	214	73 (34.1)	65 (30.4)	67 (31.3)	9 (4.2)	0.51
	Present	85	26 (30.6)	33 (38.8)	24 (28.2)	2 (2.4)	
Alcohol use	Absent	119	34 (28.6)	30 (25.2)	47 (39.5)	8 (6.7)	**0.002**
	Present	180	65 (36.1)	68 (37.8)	44 (24.4)	3 (1.7)	
**The amount of medication uses**							
0		10	5 (50.0)	3 (30.0)	2 (20.0)	0 (0)	**0.02**
1-5		65	32 (49.2)	17 (26.2)	15 (23.1)	1 (1.5)	
6-10		105	37 (35.3)	35 (33.3)	31 (29.5)	2 (1.9)	
≥11		119	25 (21.0)	43 (36.1)	43 (36.1)	8 (6.8)	
**Quality of life score**							
Physical health		60.4 ± 10.4		54.3 ± 8.6	47.3 ± 11.2	31.2 ± 12.4	**0.00**
Psychological health		64.8 ± 9.3		58.7 ± 8.3	50.2 ± 8.7	37.9 ± 14.5	**0.00**
Social relationships		66.9 ± 12.5		60.0 ± 12.9	55.4 ± 14.2	46.9 ± 10.7	**0.00**
Environmental health		60.8 ± 10.5		55.0 ± 9.2	48.2 ± 9.5	39.5 ± 9.5	**0.00**
**The amount of Social connectedness**							
Participating in social activities		2.5 ± 1.4		2.6 ± 1.3	1.8 ± 1.2	1.3 ± 0.8	**0.001**
Receiving support		1.7 ± 1.6		1.91 ± 1.7	1.6 ± 1.3	2.5 ± 1.4	0.16
Social connectedness		4.0 ± 0.9		3.9 ± 1.0	3.6 ± 1.1	2.9 ± 1.4	**0.01**

**Table 3:** Factors associated with depression by univariate and multivariate analysis.

**Contents**		**Univariate analysis**		**Multivariate analysis**			
				**Model 1**		**Model 2**	
		**Coef**	**p-value**	**Coef**	**p-value**	**Coef**	**p-value**
Age		0.37	**0.001**	0.18	**0.002**	0.13	**0.01**
Sex	Male	1		1		-	-
	Female	0.09	0.93	1.30	0.13	-	-
Education	Lower or high school	1		1		-	-
	College/Intermediated level	-0.07	0.96	0.29	0.77	-	-
	Universities or above	-1.95	0.13	0.16	0.87	-	-
Occupation	Government officer	1		1		-	-
	Worker	2.66	0.13	0.72	0.57	-	-
	Business	3.24	0.35	2.36	0.31	-	-
	Free labor	2.18	0.39	-2.25	0.22	-	-
	Housewife	-3.64	0.33	-0.04	0.99	-	-
	Others	2.82	0.59	2.23	0.54	-	-
Marital status	Single	1		1		-	-
	Married	-0.19	0.90	2.69	0.06	-	-
Living arrangement	Alone	1		1		1	
	Spouse	-1.88	0.49	-2.42	0.27	-0.42	0.82
	Children	-2.95	0.31	-3.84	0.06	-2.74	0.16
	Spouse and children	-4.95	0.07	-3.94	0.07	-1.95	0.28
	Relative and acquainted	-6.46	0.26	-11.84	**0.001**	-10.58	**0.01**
The amount of physical activities		-3.27	**0.001**	-0.87	**0.01**	-0.97	**0.01**
The amount of medication use		0.46	**0.001**	0.22	**0.01**	0.22	**0.01**
Smoking status	Absent	1		1		-	-
	Present	-0.21	0.85	2.93	**0.04**	-	-
Drinking status	Absent	1		-	-	-	-
	Present	-3.40	**0.001**	-	-	-	-
Physical health		-0.44	**0.001**	-0.15	**0.001**	-0.15	**0.001**
Psychological health		-0.50	**0.001**	-0.19	**0.001**	-0.22	**0.001**
Environmental health		-0.44	**0.001**	-0.18	**0.001**	-0.17	**0.001**
Social relationship		-0.28	**0.001**	-0.01	0.68	-	-
Social connectedness		-0.76	**0.001**	-0.21	0.18	-	-
Participate religious groups	No	1	So	-	-	1	
	Yes	0.78	0.62	-	-	2.24	**0.04**
Talk with local authority or governmental organization about problems in this community	No	1		-	-	1	
	Yes	-3.51	0.01	-	-	-1.72	**0.04**
P-value of the model				0.001		0.001	
R^2^				0.5893		0.5794	

## Data Availability

The data used to support the findings of this study are available from the corresponding author upon request.
